# Family-Based versus Unrelated Case-Control Designs for Genetic Associations

**DOI:** 10.1371/journal.pgen.0020123

**Published:** 2006-08-11

**Authors:** Evangelos Evangelou, Thomas A Trikalinos, Georgia Salanti, John P. A Ioannidis

**Affiliations:** 1 Department of Hygiene and Epidemiology, University of Ioannina School of Medicine, Ioannina, Greece; 2 Institute for Clinical Research and Health Policy Studies, Tufts University School of Medicine, Boston, Massachusetts, United States of America; 3 Biostatistics Unit, Medical Research Council, Cambridge, United Kingdom; 4 Biomedical Research Institute, Foundation for Research and Technology-Hellas, Ioannina, Greece; University of Alabama at Birmingham, United States of America

## Abstract

The most simple and commonly used approach for genetic associations is the case-control study design of unrelated people. This design is susceptible to population stratification. This problem is obviated in family-based studies, but it is usually difficult to accumulate large enough samples of well-characterized families. We addressed empirically whether the two designs give similar estimates of association in 93 investigations where both unrelated case-control and family-based designs had been employed. Estimated odds ratios differed beyond chance between the two designs in only four instances (4%). The summary relative odds ratio (ROR) (the ratio of odds ratios obtained from unrelated case-control and family-based studies) was close to unity (0.96 [95% confidence interval, 0.91–1.01]). There was no heterogeneity in the ROR across studies (amount of heterogeneity beyond chance I^2^ = 0%). Differences on whether results were nominally statistically significant (*p* < 0.05) or not with the two designs were common (opposite classification rates 14% and 17%); this reflected largely differences in power. Conclusions were largely similar in diverse subgroup analyses. Unrelated case-control and family-based designs give overall similar estimates of association. We cannot rule out rare large biases or common small biases.

## Synopsis

Different types of designs are used for the assessment of genetic associations for complex diseases. Case-control studies of unrelated people and family-based designs are the most widely used. Each has its advantages and disadvantages. This paper compares the estimates of the two types of design using a meta-analytic approach, i.e. a systematic selection of data and quantitative synthesis of results across many studies. The authors examined 93 associations where both unrelated case-control and family-based designs had been employed. Both designs gave overall similar estimates of association and the conclusions were very similar in subgroup analyses that considered various design features that might affect in theory the degree of agreement between the two designs. No heterogeneity between studies was observed. Hence, there was no consistent pattern of over-estimation or under-estimation of the probed association with one or the other design. However, one cannot exclude the possibility that rare large differences or common small differences may occur between the two designs.

## Introduction

Genetic associations for complex diseases may be probed either with case-control studies of unrelated people or with family-based designs. Both designs have advantages and disadvantages. Studies of cases and unrelated controls are the most commonly used approach; sufficiently large study populations can be readily assembled without the need to enroll also family members of the recruited participants. However, a disadvantage of this approach is that confounding due to unaccounted population admixture remains a possible threat to the validity of the obtained results [1–3]. On the other hand, family-based study designs (e.g. those including case-sibling pairs or case-parent trios) have the advantage that there is a common genetic background among the family members. Thus, the problem of population stratification is bypassed. Moreover, families tend to be more homogeneous regarding exposure to environmental factors possibly associated to the disease etiology. The main disadvantage of family-based studies, however, is that it is usually more difficult to accumulate large enough samples of well-characterized families. Therefore such studies represent the minority of investigations assessing genetic associations of complex diseases.

This problem of most appropriate versus most feasible study design may become even more pressing in the era of whole-genome strategies. Modest confounding due to population stratification may create unacceptable noise in the search for significant associations across the genome. Conversely, sample sizes need to be large enough to avoid type I error both in the screening process, as well as in the validation of what are likely to be modest genetic effects [4]. Of course, increasing sample size alone is not guaranteed to control type I error. Association studies, regardless of design, may be further confounded by genotyping error, misclassification of phenotypes and confounding by unmeasured or poorly measured environmental factors.

Strong views have been expressed on the relative merits of and preference for family-based versus unrelated controls designs [2,5]. A number of approaches have been proposed to try to detect and account for population stratification in population based studies [6–11]. Methods have also been developed to merge estimates of association from the two types of design [12,13]. Moreover, in an effort to maximize efficiency, several investigators have also proposed methods for hybrid designs that utilize data from both types of study designs [14–16]. Besides theoretical considerations, it would be interesting to obtain some empirical data on the extent to which these designs agree or disagree with each other. These data could be derived from investigations where both types of study designs were used to answer the same question on a postulated gene-disease association. We used a meta-analysis approach, i.e. a systematic selection of data and quantitative synthesis of results across many studies.

## Results

### Eligible Data

**Figure 1:**
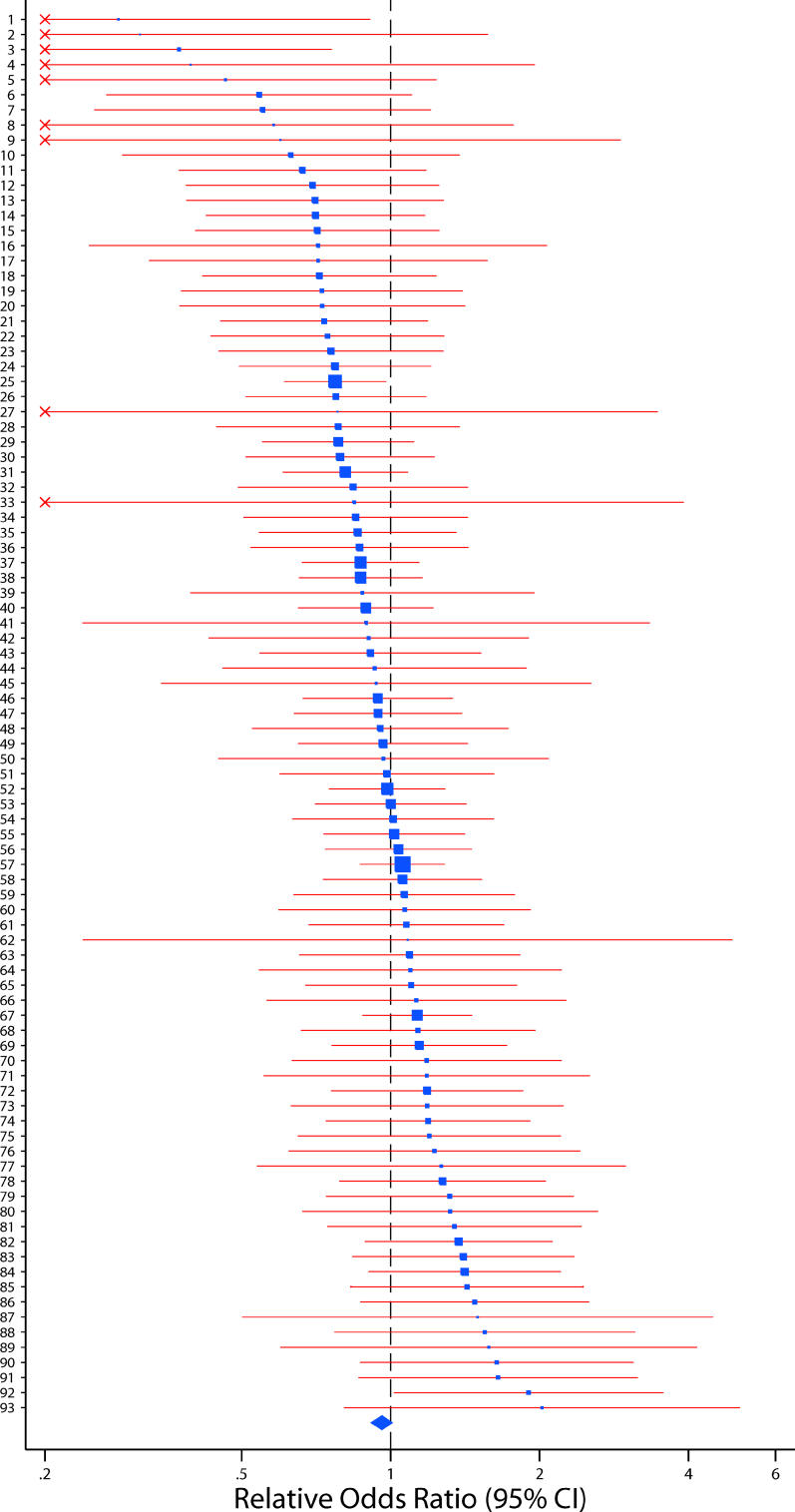
ROR and 95% CIs for Each Comparison of an Unrelated Case-Control Study versus Family-Based Study Odds ratios have been coined in such a way so that the summary OR of the two designs would be >1. Also shown are the summary ROR and its 95% CIs (diamond). Size of the boxes represents the weight of each study i which is calculated by 
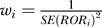
 . ID numbers correspond to Table 1. The crosses at the end of bars mean that the 95% CI extends beyond the shown range.

We analyzed a total of 93 eligible comparisons between family-based and unrelated case-control designs where both designs had been used by the same investigators to address the same postulated gene-disease association (Table 1, Dataset S1, Text S1, Figure 1). The median sample size for the unrelated case-control study was 434 (interquartile range, 280–691), while the median number of transmitted plus non-transmitted alleles for the family-based studies was 79 (interquartile range, 54–133).

**Table 1:**
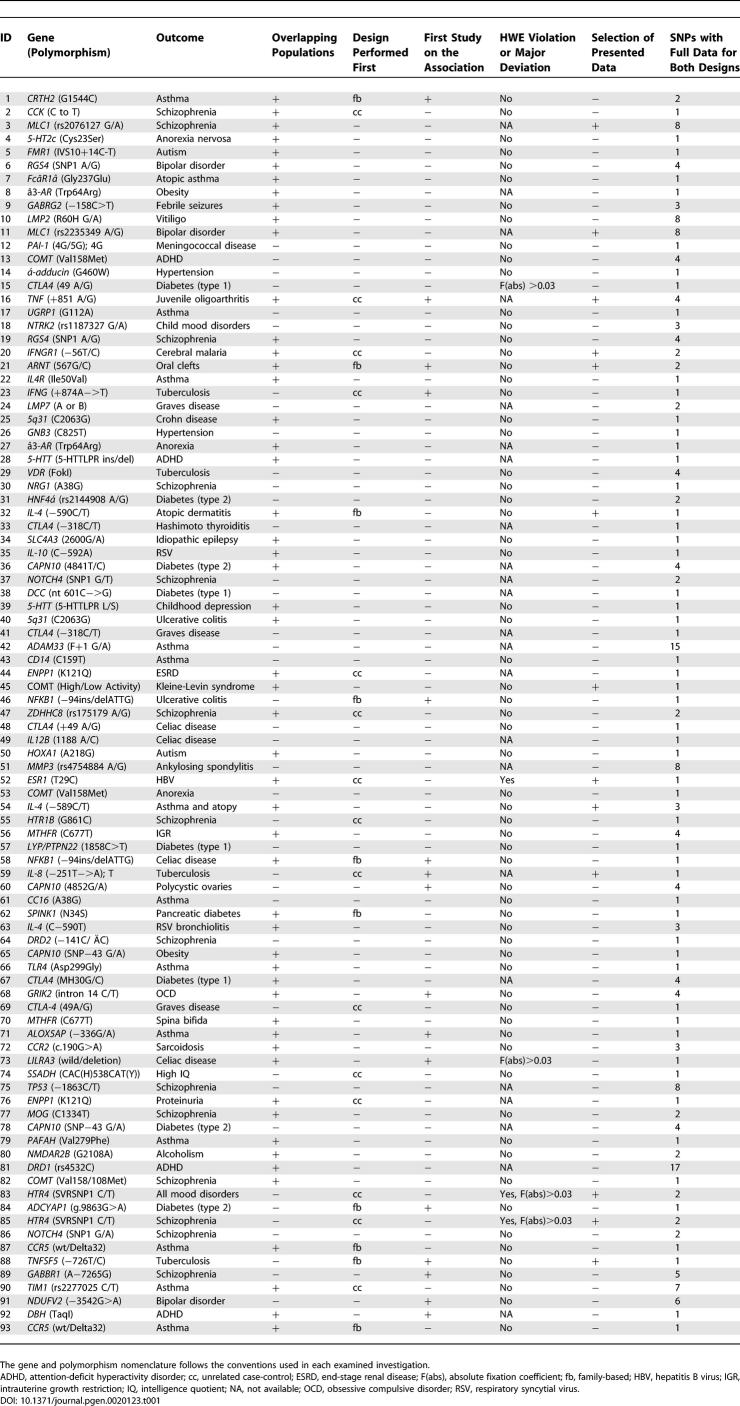
Evaluated Comparisons of Family-Based versus Unrelated Case-Control Designs

In these 93 comparisons, the populations analyzed in the family-based and unrelated case-control studies overlapped (i.e. same individuals included in both family-based and case-control designs) in 47 and in 16 comparisons it was clearly stated that this was the first published investigation addressing the specific gene-disease association. Moreover, in 25 comparisons, it was clearly stated that one design was applied first (family-based *n* = 10, unrelated case-control *n* = 15) and in 15 comparisons the results for one design had been selected for presentation based on their own statistical significance (*n* = 6 [family-based *n* = 5, unrelated case-control *n* = 1]) or the statistical significance of the results obtained with the other design (*n* = 8 [family-based *n* = 5, unrelated case-control *n* = 3]). Finally, three studies violated Hardy-Weinberg equilibrium (HWE) assumption (exact test *p* < 0.05 for the distribution of genotypes in the unrelated controls), and another two studies had absolute fixation coefficients exceeding 0.03 in the unrelated controls, even though this deviation from HWE was not formally significant.

### Comparison of Genetic Effects with the Two Designs

We combined the odds ratios (OR) in the family-based design (OR^F^) and the unrelated case-control design (OR^U^) for the minor versus major allele in order to obtain a summary OR for the strength of each probed association. When this summary OR was <1, we inversed the allele contrast, so that all summary ORs would be ≥1. We then estimated the ratio of the OR^U^ over OR^F^. This relative odds ratio (ROR) reflects the difference in the effect size between the two designs. It is expected to be 1 when the two estimates agree, >1 when the family-based design gives a smaller estimate of association than the unrelated case-control design, and <1 when the opposite occurs. When the 95% confidence interval (CI) for ROR does not contain 1, then the difference between OR^F^ and OR^U^ is beyond chance at the 0.05 level of significance.

The difference between these two estimates was significant only in four (4%) probed associations (Figure 1). An evaluation of the MLC1 rs2076127 G/A polymorphism, found a strong association with schizophrenia in the family-based design, but no effect in the unrelated case-control design [17]; the same scenario was seen for the putative association of CRTH2 (G1544C) with asthma [18] and for the putative association of 5q31 C2063G with Crohn disease [19]. Conversely, an evaluation of the DBH (TaqI) polymorphism and attention deficit hyperactivity disorder persisting into adulthood found a borderline significant association in the unrelated case-control design, but no significant association with the family-based design [20]. We perused PubMed to examine whether for these four postulated associations any additional studies had been published with larger sample size for the respective study design. We did not find any larger studies for the exact specific polymorphisms and with exactly the same phenotype. Interestingly, for DBH and attention deficit hyperactivity disorder, three previous studies on children (not adults) had claimed an association in completely the opposite direction [21–23].

Despite these isolated significant discrepancies, the summary ROR estimate across all 93 associations was 0.96 (95% CI, 0.91–1.01) showing high overall agreement. There was no heterogeneity across the ROR estimates across the 93 probed associations beyond what would be expected by chance alone (I^2^ = 0). Several studies had wide 95% CIs. Such studies would tend to increase the degrees of freedom and thus may hide some between-study heterogeneity. Analyses strictly limited to the exact same “ethnic” groups in both types of design yielded an identical summary ROR estimate of 0.96 (95% CI, 0.91–1.01), and again there was no heterogeneity between the 93 ROR estimates.

Although the differences in the results of the two designs were rarely nominally significant, the exact point estimates of the OR^F^ and OR^U^ often differed substantially. In 26 comparisons (28%), the two designs estimated effects in the opposite direction (one above 1, the other below 1). In 64 comparisons (69%), the relative risk increase (OR−1) of the unrelated case-control design was less than half or more than double compared to the family-based design.

We further examined the concordance in the level of nominal statistical significance, i.e. whether both designs found significant or non-significant results at the *p* = 0.05 level of significance. We estimated the probability that the unrelated case-control design gives a significant result and family-based gives a non-significant result, and the probability of the inverse scenario. These probabilities of opposite classification were 14% (95% CI, 8%–23%) and 17% (95% CI, 10%–27%), respectively.

### Subgroup Analyses

In theory, the design, conduct, and reporting of the two types of studies may influence the degree to which they agree. Therefore, we examined whether the results obtained with the two types of designs were more or less likely to differ systematically when there were overlapping populations in the two designs; when one design had clearly been applied first; when studies claimed to be the first article on the probed association; when results were presented based on the statistical significance of their findings or the findings of the other design; when there was violation of or major deviation from HWE in the unrelated controls; or when we had selected only one association among many presented in an article; when the sample size of unrelated case-controls contained more that 1,000 alleles; and when TDT studies used families with multiple affected sibs or not. There was no suggestion that these characteristics influenced systematically the overall summary ROR in the respective subgroup analyses (Table 2).

**Table 2:**
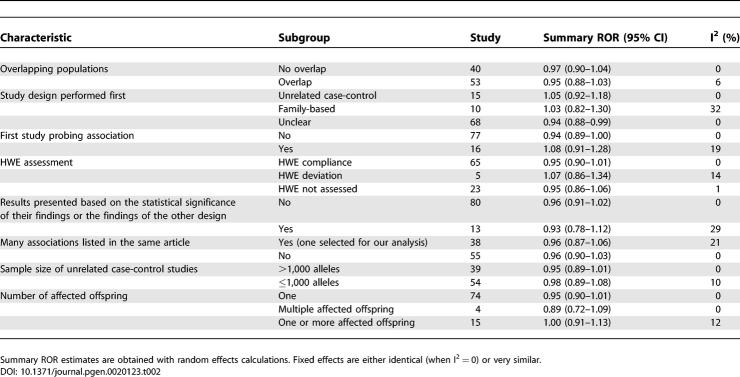
Summary RORs in Various Subgroups

### Deviation of Genetic Effect Estimates as a Function of the Amount of Data

**Figure 2:**
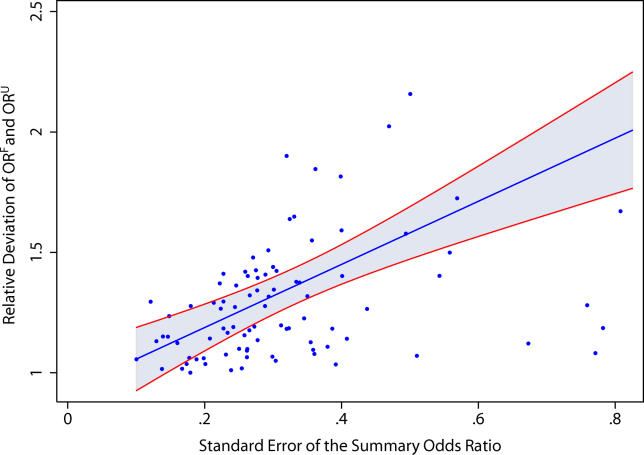
Relative Deviation of the OR with Two Designs as a Function of the Standard Error of the Summary OR The continuous bold line is the fit unweighted linear regression, and the shaded boundary presents the 95% CIs. Four outliers are not shown.

The ROR estimate is expected to fluctuate more around 1, when the data obtained by either design are more limited. Figure 2 shows the relative deviation of the two designs (ROR estimates coined to be always ≥1) as a function of their summary standard error. With a standard error of 0.29 (the median standard error observed in these 93 association datasets), on average, it is expected that the two OR estimates deviate on average 1.27-fold (95% CI, 1.00–1.66). When the standard error is halved (0.145), on average the two OR estimates are expected to deviate only 1.12-fold (95% CI, 1.00–1.37). Nevertheless, this is just an average estimate and some points, especially among the smaller studies, did not fit very well to this regression.

## Discussion

Unrelated case-control and family based-designs gave overall similar estimates of association. This means that there was no consistent pattern of over-estimation or under-estimation of the probed association with one or the other design. One might wonder whether family-based studies give much larger estimates than unrelated case-control studies in some occasions, and much smaller estimates in other occasion, but on average these differences cancel out. However, the absence of heterogeneity that we observed does not support this claim. Our analyses were more consistent with the interpretation that typically there is agreement in the estimates obtained by the two designs. Our findings should be interpreted cautiously given the small sample sizes in several of these studies.

Considerable differences in the OR point estimates between the two types of design are common and they reflect mostly the uncertainty that accompanies the estimates of small studies. If inferences are made categorically for the presence or not of formal statistical significance, discrepancies between the two study designs are common and the same applies, when inferences are made based on the magnitude of the point estimates of the genetic effects, if the uncertainty thereof, is ignored. Power deficit is a major concern for small studies and this is a greater concern for family-based designs [24], where getting sufficiently large numbers of pedigrees is not easy. While ingenious designs may improve efficiency [25] making claims for the presence or absence of an association with sparse data would be precarious with either study design. This is a major concern currently as whole genome association approaches are performed and investigators may try to employ both designs in the discovery and replication process [26]. With small sample sizes, many important genetic variants may be missed. Most of the pursued genetic associations are likely to have ORs in the range of 1.2–1.6 [4]. Given the sample sizes used in genetic association studies to-date, the average chance deviations in the estimated effects between the two designs are well in this range. Unless sample sizes increase, true signals may be buried in the noise due to chance. Simulation studies in high-throughout situations also concord that unrelated case-control designs are very powerful compared with the more laborious family-based collections [27].

We should acknowledge that with large studies of many thousands of cases and controls, even modest stratification problems may yield spuriously formally statistically significant results. Since most genome-wide approaches use formal significance rather than effect sizes to select genes for further testing, modest errors could create considerable problems. Therefore our findings should not be interpreted as evidence that unrelated case-control studies may be designed without careful standards and proper attention regarding the recruitment of the study population. Furthermore, although not common, confounding of substantial magnitude may occur sometimes even within so-called “racial” or even “ethnic” descent groups. This would not be captured by our analyses. With such confounding, even careful matching at the ethnicity level would not suffice. Our analysis suggests that large confounding effects are not common, but we cannot rule out rare large confounding effects or common small confounding effects. The latter could still bias well-powered studies using small alpha levels, a situation that is increasingly demanded in current genome wide association studies.

We should also caution that some researchers may preferentially report associations that show similar and confirmatory results with the two designs. However, our protocol excluded upfront studies where such selection was stated to have been applied. We cannot exclude the possibility that an investigator may be less likely to publish studies that found very different results in the two types of design, whereas two independent investigators may not have the same problem. Unfortunately, by default, unpublished data cannot be retrieved to see how and to what extent they might influence our conclusions. Selection choices may not be stated at all in the published reports. This publication bias would tend to increase concordance in the examined sample of investigations. However, publication bias may also decrease the overall observed concordance, if some studies with highly concordant, but “negative” results with both designs are not published [28].

We also performed subgroup analyses considering a wide range of other more subtle selection features that may in theory affect the extent of agreement between the two designs. Reassuringly, the two designs gave consistent results in all subgroup analyses. Finally, it is possible that discrepancies may be greater when the two designs have been applied by different teams of investigators. However, this concern would apply also to studies with the same design performed by different investigators.

Overall, our analysis suggests that despite the dangers of population stratification [29,30], on average, unrelated case-control designs give similar results to family-based designs. Of note, none of the 93 unrelated case-control studies analyzed here had used genomic control or other proposed methods [6–11], which in theory might have decreased further the danger of population stratification. However, this average agreement does not decrease the need to design and conduct these studies very carefully and to take all the necessary steps to avoid bias. Bias with resulting discrepancies may manifest with either false positives or true positives with biased effect estimates. Bias could be due to suboptimal population sampling, phenotype misclassification, genotyping error, confounding due to other sources, poor matching or overmatching, and selective reporting [2,31,22]. Most of these problems apply also to family-based designs [33], so these studies should not be considered immune to bias.

Allowing for these caveats, both types of design can yield useful and complementary information. Methods to improve efficiency of design and combination of data from both designs are welcome [12–16] and such methods should be tested more widely in the field. The applicability of methods of adjustment for population stratification also needs further empirical study [6–11]. However, the main problem apparently is not the lack of concordance between family-based and unrelated case-control studies, but the large uncertainty accompanying the estimates of small studies with large standard errors. Small studies are likely to suffer also more biases and may be more prone to selective reporting and publication bias [34–37]. Increasing the sample size of the available evidence should be a priority in complex disease genetics [31]. Large-scale studies and collaborative enterprises [38,39] should consider both types of designs, and may help reduce the replication uncertainty for genetic associations.

## Materials and Methods

### Eligible studies and search strategy.

We considered published studies that examined genetic associations for the same polymorphisms using both family-based and unrelated case-control designs in the same article. Eligible studies were those where we could extract or compute the allele-based log-odds ratio and its variance for at least one association with a phenotype that had been probed with both designs. For consistency, we focused on biallelic markers and binary phenotypes. We excluded studies where data had been obtained for many genetic markers and/or phenotypes, but the results had been selected for presentation based on the concordance of the two designs. We excluded studies considering microsatellites, non-biallelic markers and continuous traits; and non-English language articles.

We searched PubMed using the terms “transmission disequilibrium test,” “TDT,” “STDT,” “PDT,” “Sib-TDT,” “ETDT,” “RC-TDT,” or “family based,” combined with the terms “unrelated” or “case-control.” The search was last updated on 31 July 2005, and 2,151 items were retrieved and screened for eligibility: 1,993 could be excluded from inspecting the title and abstract, and 158 were examined in full-text for eligibility. A total of 84 articles were eligible; one gene-phenotype association was systematically selected for each of them, except for nine articles where two very different phenotypes were addressed.

For eligible reports, when data were available for the comparison of the two designs for two or more polymorphisms, we selected the one that was first mentioned in the text. We set this rule to avoid subjectivity in the selection of the polymorphism to be analyzed and to avoid having many correlated data stemming from the same compared study groups. When two or more entirely different phenotypes were examined in the same article, we considered each one of them separately.

### Databases.

We recorded the numbers in the 2 × 2 table of each analyzable unrelated case-control design for an allele-based analysis (only this would be feasible for the family-based designs). The OR^U^ was estimated as the ratio of the products of the two diagonals and the variance of its natural logarithm was estimated by the sum of the inverse of the four cells of the 2 × 2 table. We also recorded the number of transmitted (T) and non-transmitted (NT) alleles in the respective family-based design. The OR^F^ was estimated as the ratio of T over NT and the variance of its natural logarithm was estimated by the sum 1/T + 1/NT. For studies where this information was not directly available, we extracted information in order to calculate OR^F^ and OR^U^ from other presented data (*p*-values, chi-square statistics, number of informative transmissions, proportion transmitted, odds ratio and 95% CIs). OR estimates were first derived for the minor versus major allele.

In 18 studies that had used data from people of different “racial/ethnic” descent, the OR was estimated first in each “racial/ethnic” subgroup and data were then combined for each design across the available “racial/ethnic” subgroups using stratified analyses (Mantel-Haenszel method) to obtain a single OR estimate per design.

### Summary OR and ROR.

Suppose the frequency of alleles in cases and controls in the unrelated case-control study are 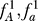 and 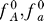 respectively. Taking allele *a* as the risk allele, the odds ratio of disease risk can be estimated by 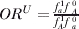 . The standard error of the natural logarithm of OR is estimated by *SE^U^* =
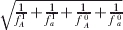
 . For TDT studies, if *T* is the number of transmitted high risk alleles and *NT* the umber of non-trasmitted alleles the odds ratio of disease risk can be estimated by 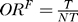 . The standard error of the natural logarithm is estimated by *SE^F^* =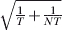 .We combined the OR^F^ and OR^U^ for the minor allele for each association in order to obtain a summary OR. This summary OR was estimated as the weighted sum of the OR^F^ and OR^U^ using the natural logarithms of the two ORs and their inverse variances as weights. For consistency, all summary ORs were then coined to be ≥1.00. This means that when the summary OR was <1 using the minor versus major allele contrast, then we used the major versus minor allele contrast instead for both the unrelated case-control and family-based data. The purpose of coining the summary OR to be ≥1.00 was to make this metric show consistently the strength of the association, regardless of whether the minor allele might be protective or conferring susceptibility. This allowed to evaluate whether unrelated case-controls consistently suggests a less strong gene-outcome association compared to family-based designs or vice versa.

The ratio of the OR^U^ and OR^F^ was calculated to obtain the ROR [26] for each probed association according to the allele contrast that yielded summary OR ≥1.00. The variance of the natural logarithm of the ROR is the sum of the variances of the natural logarithms of OR^F^ and OR^U^.

The natural logarithms of the ROR estimates were combined to obtain the summary ROR [40,41] using fixed and random effects calculations [42,43]. In fixed effects calculations it is assumed that the true effect of risk allele is the same value in each study, whereas in random effects calculations the risk allele effects for the individual studies are assumed to vary around some overall average effect. Between-study heterogeneity in the ROR estimates was quantified with the I^2^ statistic which is calculated by I^2^ = 100%(Q−df)/Q, where Q is the Cochran's heterogeneity statistic and df the degrees of freedom [44]. I^2^ ranges between 0% and 100% and estimates the amount of heterogeneity that is beyond chance. Heterogeneity is considered low, moderate, large and very large for I^2^ values of 1%–24%, 25%–49%, 50%–74%, and 75% or higher, respectively. In the absence of any heterogeneity, fixed and random effects estimates coincide.

### Measures of agreement and disagreement of the two types of design.

The main analysis examined whether the summary ROR is different from 1. The summary ROR provides an estimate of the average deviation between the odds ratios in the two study designs, i.e. whether studies with unrelated case-controls provide consistently stronger (ROR >1) or consistently weaker (ROR <1) associations than family-based studies. In addition, we evaluated whether, for specific studies, the 95% CIs of the ROR excluded 1, meaning that the results of the two types of design differed beyond chance at *p* = 0.05 level of significance.

Identifying a statistically significant difference does not depend only on the magnitude of the difference, but also on the power of the compared study designs, since very small studies may give very different point estimates, but with very large uncertainty. Therefore, we also evaluated whether the OR^F^ and OR^U^ were in the same direction (both above 1 or both below 1) and whether the magnitude of the relative risk increase (OR−1) of the unrelated case-control design was less than half or more than double compared to the family-based design. Differences in direction are important for inferences, but estimates in the opposite direction may still be very close and not incompatible with each other. The OR−1 comparison focuses on the magnitude of the difference, but does not address its statistical significance. Finally, we evaluated differences in the level of formal statistical significance, i.e. what is the probability that one design might give non-significant results, when the other design gives significant results at the 0.05 level of significance. These complementary approaches have been previously used in the comparison of effect sizes from different studies in the medical sciences [43,45,46].

### Subgroup analyses.

We recorded whether one design had clearly been applied first and the other was performed at a second stage. We also recorded various characteristics that may influence the observed concordance between the two types of designs. We noted whether there was any stated overlap between populations for each design and whether this was claimed to be the first article on this association. In theory, concordance may be larger when there is overlap in populations and first studies may also be biased towards presenting concordant results. Moreover, we recorded whether results were generated or selected for presentation based on their statistical significance, or the significance of the results of the other design; in theory, concordance may then be smaller due to regression to the mean and the winner's curse phenomenon [47]. We recorded whether the distribution of genotypes of the unrelated controls showed significant deviation from HWE based on an exact test and whether there was large deviation (fixation coefficient >0.03 in absolute value) [48] regardless of the statistical significance of the deviation. The fixation coefficient is calculated by

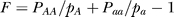
where *P_AA_* and *P_aa_* are the proportions of the homozygotes and *p_A_* and *p_a_* are the proportions of the corresponding alleles. The coefficient takes values from 1 to −1 depending on the extent of excess or deficit of homozygotes compared with the proportions expected under the Hardy-Weinberg law [48]. We finally recorded large unrelated case-control studies (>1,000 alleles) and family-based studies including multiple affected offspring. All of these characteristics were evaluated in subgroup analyses where the summary ROR was estimated separately for studies fulfilling or not each of these characteristics.

### Regression.

We examined with a linear regression, the dependence of the absolute value of the natural logarithm of the ROR on the standard error of the summary ROR (square root of the variance). The regression shows the magnitude of the absolute deviation in the OR estimates obtained with the two designs as a function of the amount of data. Although there is a certain amount of structural correlation between ROR and its standard error, this would not affect considerably the slope and 95% CIs for the regression.

## Supporting Information

Dataset S1 Raw Data of the 93 Eligible Comparisons Used for the Analysis (44 KB XLS)

Text S1 Reference List of All Eligible Articles (47 KB DOC)

## Abbreviations

CI: confidence interval
HWE: Hardy-Weinberg equilibrium
OR: odds ratio
ROR: relative odds ratio
