# Supplementary material for: Family-Based versus Unrelated Case-Control Designs for Genetic Associations
Source: PLoS Genet. 2006 Aug 11;2(8):e123. doi: 10.1371/journal.pgen.0020123 (PMC1534078; doi:10.1371/journal.pgen.0020123)
Supplement: Supplementary file 1 [file pgen.0020123.se001.doc]

References

1.	Huang, J.L. et al. Sequence variants of the gene encoding chemoattractant receptor expressed on Th2 cells (CRTH2) are associated with asthma and differentially influence mRNA stability. *Hum Mol Genet* **13**, 2691-7 (2004).

2.	Wang, Z., Wassink, T., Andreasen, N.C. & Crowe, R.R. Possible association of a cholecystokinin promoter variant to schizophrenia. *Am J Med Genet* **114**, 479-82 (2002).

3.	Verma, R. et al. MLC1 gene is associated with schizophrenia and bipolar disorder in Southern India. *Biol Psychiatry* **58**, 16-22 (2005).

4.	Hu, X. et al. Association of the 5-HT2c gene with susceptibility and minimum body mass index in anorexia nervosa. *Neuroreport* **14**, 781-3 (2003).

5.	Vincent, J.B. et al. Association and transmission analysis of the FMR1 IVS10 + 14C-T variant in autism. *Am J Med Genet B Neuropsychiatr Genet* **125**, 54-6 (2004).

6.	Cordeiro, Q. et al. Association and linkage analysis of RGS4 polymorphisms with schizophrenia and bipolar disorder in Brazil. *Genes Brain Behav* **4**, 45-50 (2005).

7.	Ishizawa, M. et al. No association between atopic asthma and a coding variant of Fc epsilon R1 beta in a Japanese population. *J Hum Genet* **44**, 308-11 (1999).

8.	Hinney, A. et al. Beta 3-adrenergic-receptor allele distributions in children, adolescents and young adults with obesity, underweight or anorexia nervosa. *Int J Obes Relat Metab Disord* **21**, 224-30 (1997).

9.	Nakayama, J. et al. Failure to find causal mutations in the GABA(A)-receptor gamma2 subunit (GABRG2) gene in Japanese febrile seizure patients. *Neurosci Lett* **343**, 117-20 (2003).

10.	Casp, C.B., She, J.X. & McCormack, W.T. Genes of the LMP/TAP cluster are associated with the human autoimmune disease vitiligo. *Genes Immun* **4**, 492-9 (2003).

11.	Haralambous, E. et al. Role of functional plasminogen-activator-inhibitor-1 4G/5G promoter polymorphism in susceptibility, severity, and outcome of meningococcal disease in Caucasian children. *Crit Care Med* **31**, 2788-93 (2003).

12.	Qian, Q. et al. Family-based and case-control association studies of catechol-O-methyltransferase in attention deficit hyperactivity disorder suggest genetic sexual dimorphism. *Am J Med Genet B Neuropsychiatr Genet* **118**, 103-9 (2003).

13.	Ju, Z. et al. Alpha-adducin gene polymorphism is associated with essential hypertension in Chinese: a case-control and family-based study. *J Hypertens* **21**, 1861-8 (2003).

14.	Fajardy, I. et al. CTLA-4 49 A/G dimorphism and type 1 diabetes susceptibility: a French case-control study and segregation analysis. Evidence of a maternal effect. *Eur J Immunogenet* **29**, 251-7 (2002).

15.	Zeggini, E. et al. Linkage and association studies of single-nucleotide polymorphism-tagged tumor necrosis factor haplotypes in juvenile oligoarthritis. *Arthritis Rheum* **46**, 3304-11 (2002).

16.	Batra, J., Niphadkar, P.V., Sharma, S.K. & Ghosh, B. Uteroglobin-related protein 1(UGRP1) gene polymorphisms and atopic asthma in the Indian population. *Int Arch Allergy Immunol* **136**, 1-6 (2005).

17.	Adams, J.H. et al. Association study of neurotrophic tyrosine kinase receptor type 2 (NTRK2) and childhood-onset mood disorders. *Am J Med Genet B Neuropsychiatr Genet* **132**, 90-5 (2005).

18.	Koch, O. et al. IFNGR1 gene promoter polymorphisms and susceptibility to cerebral malaria. *J Infect Dis* **185**, 1684-7 (2002).

19.	Kayano, S. et al. Significant association between nonsyndromic oral clefts and arylhydrocarbon receptor nuclear translocator (ARNT). *Am J Med Genet A* **130**, 40-4 (2004).

20.	Noguchi, E. et al. No association between atopy/asthma and the ILe50Val polymorphism of IL-4 receptor. *Am J Respir Crit Care Med* **160**, 342-5 (1999).

21.	Rossouw, M., Nel, H.J., Cooke, G.S., van Helden, P.D. & Hoal, E.G. Association between tuberculosis and a polymorphic NFkappaB binding site in the interferon gamma gene. *Lancet* **361**, 1871-2 (2003).

22.	Heward, J.M. et al. Association of the large multifunctional proteasome (LMP2) gene with Graves' disease is a result of linkage disequilibrium with the HLA haplotype DRB1*0304-DQB1*02-DQA1*0501. *Clin Endocrinol (Oxf)* **51**, 115-8 (1999).

23.	Mirza, M.M. et al. Genetic evidence for interaction of the 5q31 cytokine locus and the CARD15 gene in Crohn disease. *Am J Hum Genet* **72**, 1018-22 (2003).

24.	Huang, X. et al. Lack of association between the G protein beta3 subunit gene and essential hypertension in Chinese: a case-control and a family-based study. *J Mol Med* **81**, 729-35 (2003).

25.	Langley, K. et al. No evidence of association of two 5HT transporter gene polymorphisms and attention deficit hyperactivity disorder. *Psychiatr Genet* **13**, 107-10 (2003).

26.	Bornman, L. et al. Vitamin D receptor polymorphisms and susceptibility to tuberculosis in West Africa: a case-control and family study. *J Infect Dis* **190**, 1631-41 (2004).

27.	Hong, C.J. et al. Case-control and family-based association studies between the neuregulin 1 (Arg38Gln) polymorphism and schizophrenia. *Neurosci Lett* **366**, 158-61 (2004).

28.	Weedon, M.N. et al. Common variants of the hepatocyte nuclear factor-4alpha P2 promoter are associated with type 2 diabetes in the U.K. population. *Diabetes* **53**, 3002-6 (2004).

29.	Kawashima, T. et al. Linkage and association of an interleukin 4 gene polymorphism with atopic dermatitis in Japanese families. *J Med Genet* **35**, 502-4 (1998).

30.	Heward, J.M. et al. No evidence for allelic association of a human CTLA-4 promoter polymorphism with autoimmune thyroid disease in either population-based case-control or family-based studies. *Clin Endocrinol (Oxf)* **49**, 331-4 (1998).

31.	Sander, T. et al. Association of the 867Asp variant of the human anion exchanger 3 gene with common subtypes of idiopathic generalized epilepsy. *Epilepsy Res* **51**, 249-55 (2002).

32.	Hoebee, B. et al. Influence of promoter variants of interleukin-10, interleukin-9, and tumor necrosis factor-alpha genes on respiratory syncytial virus bronchiolitis. *J Infect Dis* **189**, 239-47 (2004).

33.	Evans, J.C. et al. Studies of association between the gene for calpain-10 and type 2 diabetes mellitus in the United Kingdom. *Am J Hum Genet* **69**, 544-52 (2001).

34.	Fan, J.B. et al. A family-based and case-control association study of the NOTCH4 gene and schizophrenia. *Mol Psychiatry* **7**, 100-3 (2002).

35.	Hall, R.J. et al. The deleted in colorectal carcinoma (DCC) gene 201 R --> G polymorphism: no evidence for genetic association with autoimmune disease. *Eur J Hum Genet* **11**, 840-4 (2003).

36.	Nobile, M. et al. A case-control and family-based association study of the 5-HTTLPR in pediatric-onset depressive disorders. *Biol Psychiatry* **56**, 292-5 (2004).

37.	Werner, M. et al. Asthma is associated with single-nucleotide polymorphisms in ADAM33. *Clin Exp Allergy* **34**, 26-31 (2004).

38.	Sharma, M. et al. Suggestive evidence of association of C-159T functional polymorphism of the CD14 gene with atopic asthma in northern and northwestern Indian populations. *Immunogenetics* **56**, 544-7 (2004).

39.	Canani, L.H. et al. Polymorphism in ecto-nucleotide pyrophosphatase/phosphodiesterase 1 gene (ENPP1/PC-1) and early development of advanced diabetic nephropathy in type 1 diabetes. *Diabetes* **51**, 1188-93 (2002).

40.	Dauvilliers, Y. et al. Kleine-Levin syndrome: an autoimmune hypothesis based on clinical and genetic analyses. *Neurology* **59**, 1739-45 (2002).

41.	Karban, A.S. et al. Functional annotation of a novel NFKB1 promoter polymorphism that increases risk for ulcerative colitis. *Hum Mol Genet* **13**, 35-45 (2004).

42.	Chen, W.Y. et al. Case-control study and transmission disequilibrium test provide consistent evidence for association between schizophrenia and genetic variation in the 22q11 gene ZDHHC8. *Hum Mol Genet* **13**, 2991-5 (2004).

43.	Mora, B. et al. CTLA-4 +49 A/G dimorphism in Italian patients with celiac disease. *Hum Immunol* **64**, 297-301 (2003).

44.	Louka, A.S. et al. The IL12B gene does not confer susceptibility to coeliac disease. *Tissue Antigens* **59**, 70-2 (2002).

45.	Conciatori, M. et al. Association between the HOXA1 A218G polymorphism and increased head circumference in patients with autism. *Biol Psychiatry* **55**, 413-9 (2004).

46.	Jin, L. et al. Lack of association of matrix metalloproteinase 3 (MMP3) genotypes with ankylosing spondylitis susceptibility and severity. *Rheumatology (Oxford)* **44**, 55-60 (2005).

47.	Deng, G. et al. Association of estrogen receptor alpha polymorphisms with susceptibility to chronic hepatitis B virus infection. *Hepatology* **40**, 318-26 (2004).

48.	Gabrovsek, M. et al. Combined family trio and case-control analysis of the COMT Val158Met polymorphism in European patients with anorexia nervosa. *Am J Med Genet B Neuropsychiatr Genet* **124**, 68-72 (2004).

49.	Beghe, B. et al. Polymorphisms in the interleukin-4 and interleukin-4 receptor alpha chain genes confer susceptibility to asthma and atopy in a Caucasian population. *Clin Exp Allergy* **33**, 1111-7 (2003).

50.	Duan, S. et al. No association between the serotonin 1B receptor gene and schizophrenia in a case-control and family-based association study. *Neurosci Lett* **376**, 93-7 (2005).

51.	Infante-Rivard, C. et al. Absence of association of thrombophilia polymorphisms with intrauterine growth restriction. *N Engl J Med* **347**, 19-25 (2002).

52.	Smyth, D. et al. Replication of an association between the lymphoid tyrosine phosphatase locus (LYP/PTPN22) with type 1 diabetes, and evidence for its role as a general autoimmunity locus. *Diabetes* **53**, 3020-3 (2004).

53.	Ma, X. et al. Association between interleukin-8 gene alleles and human susceptibility to tuberculosis disease. *J Infect Dis* **188**, 349-55 (2003).

54.	Haddad, L. et al. Variation within the type 2 diabetes susceptibility gene calpain-10 and polycystic ovary syndrome. *J Clin Endocrinol Metab* **87**, 2606-10 (2002).

55.	Sharma, S. & Ghosh, B. Association of an intragenic microsatellite marker in the CC16 gene with asthma in the Indian population. *J Hum Genet* **49**, 677-83 (2004).

56.	Hassan, Z. et al. SPINK1 is a susceptibility gene for fibrocalculous pancreatic diabetes in subjects from the Indian subcontinent. *Am J Hum Genet* **71**, 964-8 (2002).

57.	Hoebee, B. et al. Association of severe respiratory syncytial virus bronchiolitis with interleukin-4 and interleukin-4 receptor alpha polymorphisms. *J Infect Dis* **187**, 2-11 (2003).

58.	Li, T. et al. Case-control, haplotype relative risk and transmission disequilibrium analysis of a dopamine D2 receptor functional promoter polymorphism in schizophrenia. *Schizophr Res* **32**, 87-92 (1998).

59.	Carlsson, E., Fredriksson, J., Groop, L. & Ridderstrale, M. Variation in the calpain-10 gene is associated with elevated triglyceride levels and reduced adipose tissue messenger ribonucleic acid expression in obese Swedish subjects. *J Clin Endocrinol Metab* **89**, 3601-5 (2004).

60.	Yang, I.A. et al. Toll-like receptor 4 polymorphism and severity of atopy in asthmatics. *Genes Immun* **5**, 41-5 (2004).

61.	Zhernakova, A. et al. CTLA4 is differentially associated with autoimmune diseases in the Dutch population. *Hum Genet* **118**, 58-66 (2005).

62.	Delorme, R. et al. Frequency and transmission of glutamate receptors GRIK2 and GRIK3 polymorphisms in patients with obsessive compulsive disorder. *Neuroreport* **15**, 699-702 (2004).

63.	Heward, J.M. et al. The development of Graves' disease and the CTLA-4 gene on chromosome 2q33. *J Clin Endocrinol Metab* **84**, 2398-401 (1999).

64.	Koch, M.C., Stegmann, K., Ziegler, A., Schroter, B. & Ermert, A. Evaluation of the MTHFR C677T allele and the MTHFR gene locus in a German spina bifida population. *Eur J Pediatr* **157**, 487-92 (1998).

65.	Sayers, I. et al. Promoter polymorphism in the 5-lipoxygenase (ALOX5) and 5-lipoxygenase-activating protein (ALOX5AP) genes and asthma susceptibility in a Caucasian population. *Clin Exp Allergy* **33**, 1103-10 (2003).

66.	Valentonyte, R. et al. Study of C-C chemokine receptor 2 alleles in sarcoidosis, with emphasis on family-based analysis. *Am J Respir Crit Care Med* **171**, 1136-41 (2005).

67.	Moodie, S.J. et al. Analysis of candidate genes on chromosome 19 in coeliac disease: an association study of the KIR and LILR gene clusters. *Eur J Immunogenet* **29**, 287-91 (2002)..

68.	Plomin, R. et al. A functional polymorphism in the succinate-semialdehyde dehydrogenase (aldehyde dehydrogenase 5 family, member A1) gene is associated with cognitive ability. *Mol Psychiatry* **9**, 582-6 (2004).

69.	Ni, X. et al. Human p53 tumor suppressor gene (TP53) and schizophrenia: case-control and family studies. *Neurosci Lett* **388**, 173-8 (2005).

70.	Zai, G. et al. Genetic study of the myelin oligodendrocyte glycoprotein (MOG) gene in schizophrenia. *Genes Brain Behav* **4**, 2-9 (2005).

71.	Orho-Melander, M. et al. Variants in the calpain-10 gene predispose to insulin resistance and elevated free fatty acid levels. *Diabetes* **51**, 2658-64 (2002).

72.	Ito, S. et al. Evidence for an association between plasma platelet-activating factor acetylhydrolase deficiency and increased risk of childhood atopic asthma. *J Hum Genet* **47**, 99-101 (2002).

73.	Wernicke, C. et al. Polymorphisms in the N-methyl-D-aspartate receptor 1 and 2B subunits are associated with alcoholism-related traits. *Biol Psychiatry* **54**, 922-8 (2003).

74.	Bobb, A.J. et al. Support for association between ADHD and two candidate genes: NET1 and DRD1. *Am J Med Genet B Neuropsychiatr Genet* **134**, 67-72 (2005).

75.	Kremer, I. et al. Family-based and case-control study of catechol-O-methyltransferase in schizophrenia among Palestinian Arabs. *Am J Med Genet B Neuropsychiatr Genet* **119**, 35-9 (2003).

76.	Ohtsuki, T. et al. Association between serotonin 4 receptor gene polymorphisms and bipolar disorder in Japanese case-control samples and the NIMH Genetics Initiative Bipolar Pedigrees. *Mol Psychiatry* **7**, 954-61 (2002).

77.	Gu, H.F. Genetic variation screening and association studies of the adenylate cyclase activating polypeptide 1 (ADCYAP1) gene in patients with type 2 diabetes. *Hum Mutat* **19**, 572-3 (2002).

78.	Kaneko, N. et al. Transmission disequilibrium test and haplotype analysis of the NOTCH4 gene in Japanese patients with schizophrenia. Psychiatry Clin Neurosci 58, 199-205 (2004).

79.	McGinnis, R. et al. Further support for the association of CCR5 allelic variants with asthma susceptibility. Eur J Immunogenet 29, 525-8 (2002).

80.	Campbell, S.J. et al. Variants of the CD40 ligand gene are not associated with increased susceptibility to tuberculosis in West Africa. Immunogenetics 55, 502-7 (2003).

81.	Zai, G., King, N., Wong, G.W., Barr, C.L. & Kennedy, J.L. Possible association between the gamma-aminobutyric acid type B receptor 1 (GABBR1) gene and schizophrenia. Eur Neuropsychopharmacol 15, 347-52 (2005).

82.	Gao, P.S. et al. Genetic variants of the T-cell immunoglobulin mucin 1 but not the T-cell immunoglobulin mucin 3 gene are associated with asthma in an African American population. J Allergy Clin Immunol 115, 982-8 (2005).

83.	Washizuka, S. et al. Association of mitochondrial complex I subunit gene NDUFV2 at 18p11 with bipolar disorder in Japanese and the National Institute of Mental Health pedigrees. Biol Psychiatry 56, 483-9 (2004).

84.	Inkster, B., Muglia, P., Jain, U. & Kennedy, J.L. Linkage disequilibrium analysis of the dopamine beta-hydroxylase gene in persistent attention deficit hyperactivity disorder. Psychiatr Genet 14, 117-20 (2004).
